# An accurate interactive segmentation and volume calculation of orbital soft tissue for orbital reconstruction after enucleation

**DOI:** 10.1186/s12886-019-1260-5

**Published:** 2019-12-16

**Authors:** Qingyao Ning, Xiaoyao Yu, Qi Gao, Jiajun Xie, Chunlei Yao, Kun Zhou, Juan Ye

**Affiliations:** 1 Department of Ophthalmology, the Second Affiliated Hospital of Zhejiang University, College of Medicine, No. 88 Jiefang Road, Hangzhou, 310009 Zhejiang Province China; 2 State Key Lab of CAD & CG, Zhejiang University, No. 886 Yuhangtang Road, Hangzhou, 310058 Zhejiang Province China

**Keywords:** Orbit soft tissue, Orbital reconstruction, Computerized tomography, Tissue segmentation

## Abstract

**Background:**

Accurate measurement and reconstruction of orbital soft tissue is important to diagnosis and treatment of orbital diseases. This study applied an interactive graph cut method to orbital soft tissue precise segmentation and calculation in computerized tomography (CT) images, and to estimate its application in orbital reconstruction.

**Methods:**

The interactive graph cut method was introduced to segment extraocular muscle and intraorbital fat in CT images. Intra- and inter-observer variability of tissue volume measured by graph cut segmentation was validated. Accuracy and reliability of the method was accessed by comparing with manual delineation and commercial medical image software. Intraorbital structure of 10 patients after enucleation surgery was reconstructed based on graph cut segmentation and soft tissue volume were compared within two different surgical techniques.

**Results:**

Both muscle and fat tissue segmentation results of graph cut method showed good consistency with ground truth in phantom data. There were no significant differences in muscle calculations between observers or segmental methods (*p* > 0.05). Graph cut results of fat tissue had coincidental variable trend with ground truth which could identify 0.1cm^3^ variation. The mean performance time of graph cut segmentation was significantly shorter than manual delineation and commercial software (*p* < 0.001). Jaccard similarity and Dice coefficient of graph cut method were 0.767 ± 0.045 and 0.836 ± 0.032 for human normal extraocular muscle segmentation. The measurements of fat tissue were significantly better in graph cut than those in commercial software (*p* < 0.05). Orbital soft tissue volume was decreased in post-enucleation orbit than that in normal orbit (*p* < 0.05).

**Conclusion:**

The graph cut method was validated to have good accuracy, reliability and efficiency in orbit soft tissue segmentation. It could discern minor volume changes of soft tissue. The interactive segmenting technique would be a valuable tool for dynamic analysis and prediction of therapeutic effect and orbital reconstruction.

## Background

Orbital soft tissues, including extraocular muscles and intraorbital fat, are the important components of orbital contents, accounting for approximately 50% of orbital cavity volume [1, 2]. Volume or distribution changes of the soft tissue caused by orbital trauma and orbital relative diseases (such as orbital fracture, thyroid-associated ophthalmopathy (TAO), intraorbital tumor and so on) may lead to diplopia, restricted ocular movement and even impairment of visual function [3, 4]. Precise assessment of intraorbital soft tissue is essential for diagnosis of intraorbital disorders, effect evaluation of treatment and planning for surgical intervention [5–10].

Computerized tomography (CT) and magnetic resonance imaging (MRI) are the main examining approaches to observe intraorbital soft tissues. Clinicians could gain comprehensive and quantitative information through three-dimensional (3D) reconstruction and parameter measurements of the interested tissues in the medical images [11–13]. In this process, accurate discrimination of the target tissue and its boundary is the basic and the most important step to ensure the accuracy of subsequent quantitative analysis. Manual delineation of tissue by doctors is a traditional and standard approach in clinical practice. However, it is labor-intensive and time-consuming. Several studies have focused on segmenting the interested tissues of orbit using semiautomatic or even automatic approaches based on computer image processing techniques [14–17]. However, due to the complicated structure and the small volume of orbit, as well as the unclear boundary of soft tissues in CT images, effective and precise segmentation of intraorbital fat and extraocular muscles remains a tough task [14, 18].

Some early computer segmentation methods distinguish different regions according to different density or colour of the pixels in the image. While, Boykov et al. provided the graph cut algorithm which could take into account both the density of each pixel and the connectivity between the pixels, making the segmentation process more intellegent and accurate than before [19, 20]. In recent years, the graph cut algorithms have been applied to segment specific tissues in medical image, such as liver, artery wall and lung tumor, as well as the analysis of ocular fundus images [21–27]. The aims of this study were to introduce an interactive segmentation method based on graph cut algorithm and to evaluate the accuracy and reliability of the method for orbital soft tissue segmentation and clinical reconstruction.

## Methods

### Phantom and patient

This study was approved by the Medical Ethics Committee of the Second Affiliated Hospital of Zhejiang University and adhered to the guidelines of the Declaration of Helsinki. All patients provided informed consent before participation in the study.

To validate the segmentation method based on graph cut algorithm, we constructed an idealized orbit phantom with butter and pork tenderloin as equivalents for orbital fat and extraocular muscles, respectively, according to Regensburg’s method [14]. Human dry skull was provided by the Department of Anatomy, Zhejiang University, School of Medicine. Pork tenderloin was cut into four muscle strips with the total volume measured as 3.8 cm^3^. Butter, muscle strips and hydroxyapatite (HA) sphere prostheses were inserted into the right orbital cavity of the skull, while only butter in a solid-stated was inserted into the left orbital cavity. The volume of fat was gradually increased with the variation from 0.1 cm^3^ to 2.5 cm^3^, thus form into six orbit phantoms. All the phantoms underwent orbital CT scanning.

Ten adult patients who underwent enucleation and orbital prosthesis implantation took the orbital CT exanmination three months after surgery. As we reported previously, there were two closure methods used for hydroxyapatite (HA) sphere prosthesis implantation [28]. Of these patients, five underwent rectus end-to-end suturing procedure, and five underwent rectus orthotopic suturing procedure.

### CT protocol

Axial and coronal orbital CT scans of phantoms and patients were acquired via a multislice spiral CT (SOMATOM Sensation 16, Siemens, German). Continuous scanning with a slice thickness of 1.0 mm and a slice increment of 1.0 mm was applied. CT images were output and saved as DICOM files for further processing.

### Segmentation of orbital soft tissue in CT image

In this study, our interactive graph cut segmentation function was implemented by being plugged into an open source medical imaging computation and analysis platform called 3DMed [29]. CT images were loaded into this processing software. The unilateral orbital regions were extracted as the region of interest (ROI), and used for the subsequent identification and segmentation of intraorbital soft tissues. ROI volume data of each subject was composed of approximately 40 CT image slices with approximately 100 × 150 pixels per-slice.

**Fig. 1:**
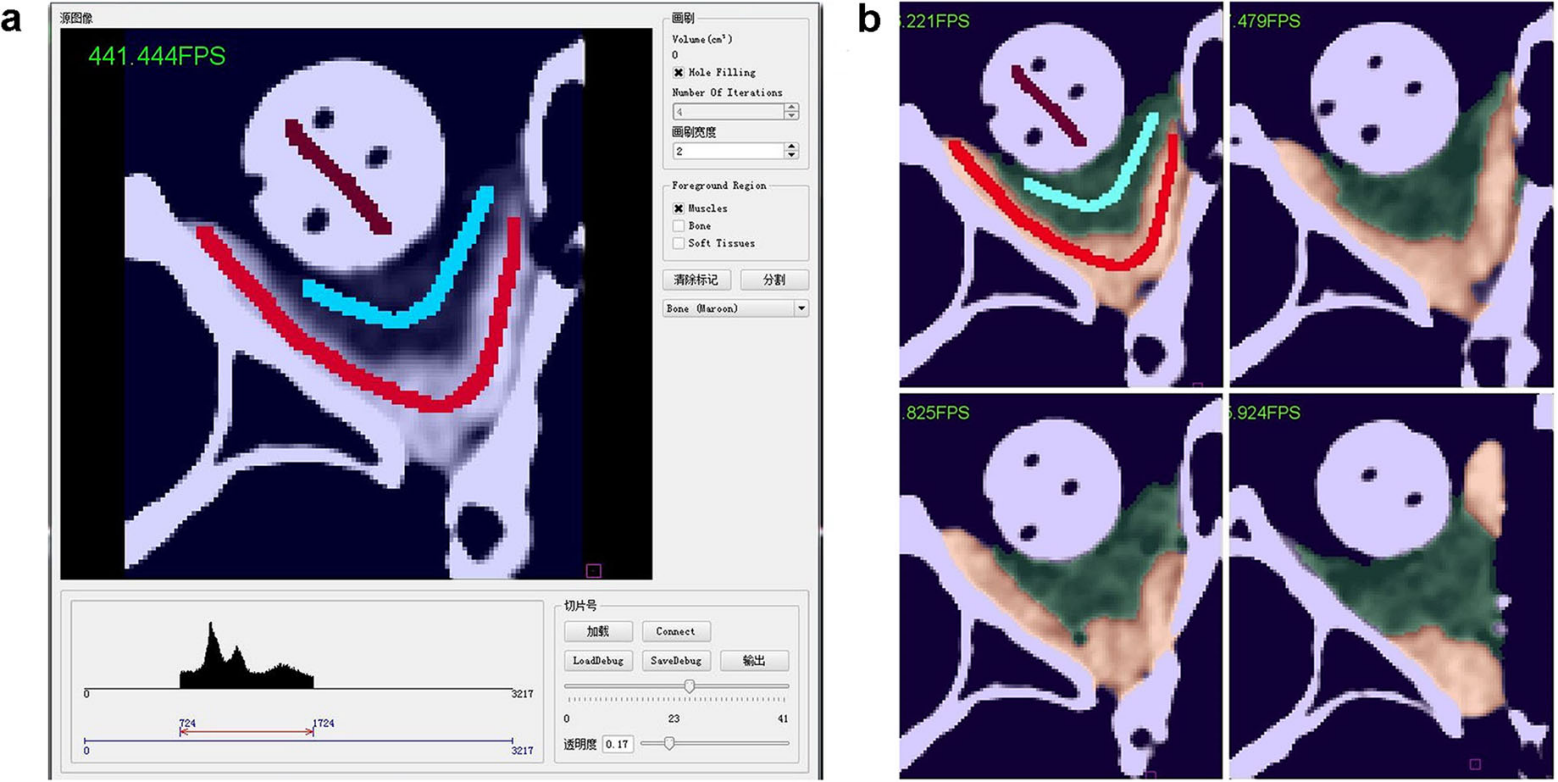
The operation interface of graph cut process and the results of orbital soft tissue segmentation. **a** shows user interface of graph cut process and the ROI of phantom CT image after volume data clipping. Here is the right side of phantom orbit. User labeled seed points of different tissues on this slice. Red line marked extraocular muscle, blue line marked orbital fat, maroon line marked bone and HA prosthesis. All the slices in ROI volume data would be segmented according to these labels. **b** shows the segmentation results of the marked slice and other slices by graph cut process. The red part shows muscle region and blue part shows fat region

We introduced lazy snapping algorithm [30] as an interactive tool between users and computer, helping users defining samples of different tissues for the following automatic tissue segmentation. In this pre-segmentation process, three brushes were designed for specific tissues in the orbit: red lines marked muscle, blue lines marked fat, and maroon lines marked bony dense structures (Fig. 1a). After some marking lines were drawn on muscle, fat and bone areas in one or several CT image slices, the computer would automatically discriminate different tissues in all slices of CT image according to the sample labels, and output the segmentation results and tissue volumes (Fig. 1b). Additional details regarding graph cut and lazy snapping algorithm are presented in Additional file 1.

### Data measurements

Extraocular muscle and intraocular fat were segmented and volume calculated by three different methods: manual delineation, commercial Mimics software (Materialise, version 17.0, Louvain, Belgium) and the graph cut method. Performance time of the methods were tracked when segmenting images of orbit phantoms. A senior ophthalmologist specialized in orbital diseases (OA) and a junior ophthalmologist (OB) participated in this study. The measurements were performed independently but concurrently by the two observers on computers with the same configuration.

### Statistical analysis

All statistical analyses were performed with SPSS software (V.18 for windows; SPSS, Chicago, Illinois, USA), and *p* < 0.05 was considered statistically significant. The measured values were presented as mean ± SD (standard deviation). Comparisons of extraocular muscle volume (MV) for intraobserver and interobserver variabilities and between different methods were performed using the Student’s paired-sample t test. Intraobserver and interobserver variability in fat volume (FV) were assessed by intraclass correlation coefficients (ICC) of a two-way mixed-effects model (0 = no agreement, 1 = perfect agreement). It was considered to be adequate if ICC was > 0.75 [31]. Jaccard similarity (JS) and Dice coefficient (DC) were used to evaluate the accuracy of the segmentation methods in human normal data [32, 33]. A value of 0 indicates that the two sets were completely dissimilar, while 1 indicates that the two sets were identical. Differences in extraocular muscle and fat volume between the two surgical methods were estimated with Student’s independent-sample t test.

## Results

### Orbital phantom CT image segmentation results

#### Extraocular muscle calculation

Every phantom dataset was repeatedly segmented by each observer using graph cut method. The MV calculated the first time by OA was 3.7571 ± 0.0563 cm^3^, and the value obtained the second time was 3.8009 ± 0.0059 cm^3^. Intraobserver variability was with no significant differences (*p* = 0.115, paired t test). The measurements of OB were 3.7686 ± 0.0544 cm^3^ and 3.7845 ± 0.0348 cm^3^, respectively (*p* = 0.560, paired t test).

**Fig. 2:**
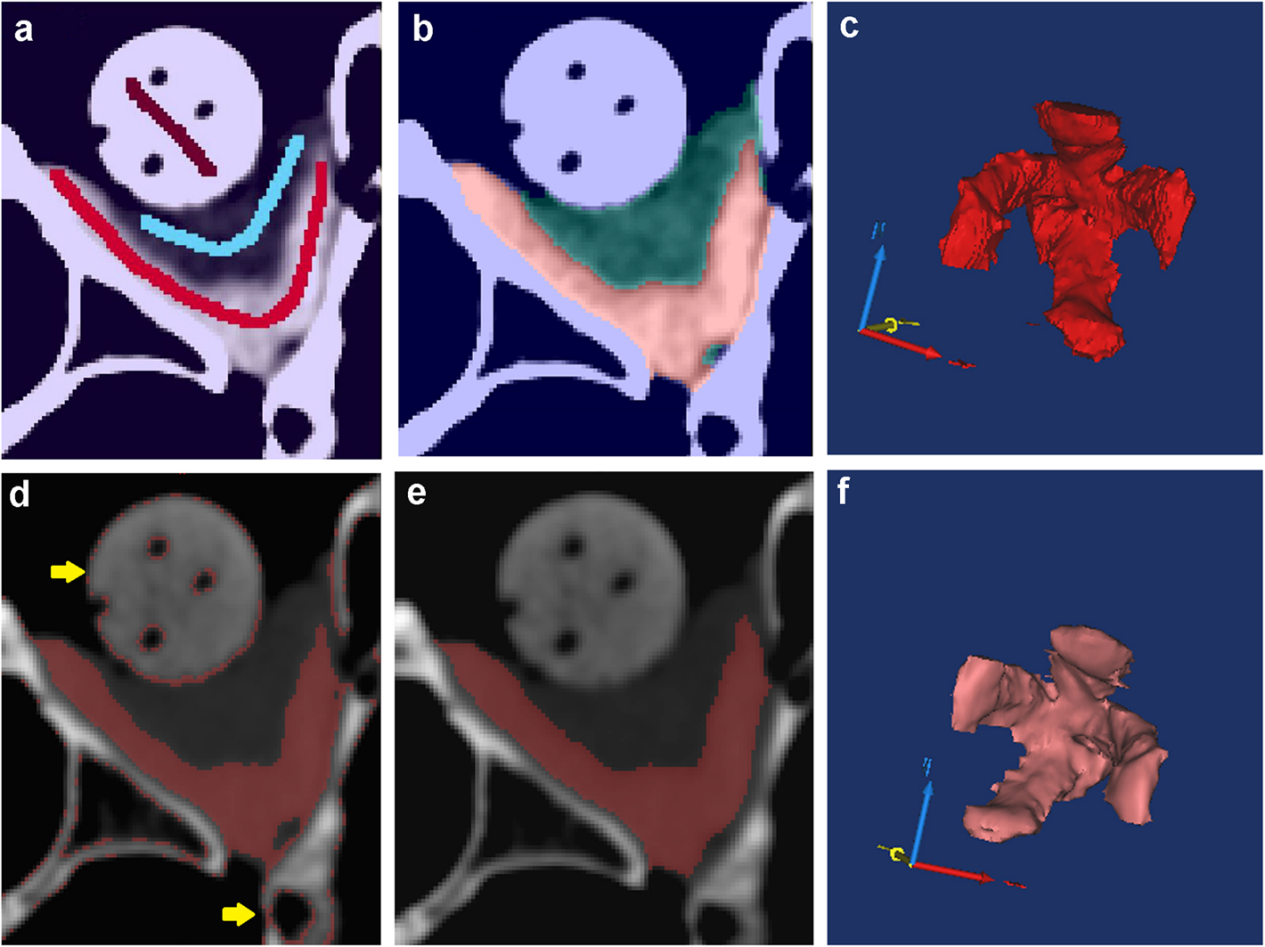
Segmentation results and 3D reconstruction of phantom extraocular muscle by graph cut method and Mimics software. **a**-**c** show the graph cut segmentation results and muscle reconstruction without manual modification. **d**-**f** show the results of Mimics segmentation. In graph (**d**), the red part shows primary segmented region which included a lot of redundant regions outside the muscle as the yellow arrow indicated. **e** shows the modified result after observers erasing the redundant regions manually. **f** is the 3D visualization of the segmented and modified muscle in Mimics software

The MV measurements of two observers using three different methods were shown in Table 1. There was no significant difference between the observers when using the graph cut method (*p* = 0.895, paired t test). There were also no significant differences among the three methods when results were obtained by the same observer (*P* = 0.087–0.770, paired t test). Segmentation results and muscle 3D reconstruction of graph cut method and Mimics software were showed in Fig. 2. In Mimics software, the primary segmentation produces into redundant results at the boundaries of tissues that need to be erased manually by the observers. The final volume of muscle was calculated after this modification.

**Table 1:** The interobserver variability (Student’s paired sample t-test) and percentage differences of three different segmentation methods for MV calculation in orbital phantom data

	MV of OA (cm^3^, *n* = 6 or 12*)	MV of OB (cm^3^, n = 6 or 12*)	T value	*P* value	Total MV (cm^3^, n = 12 or 24*)	Percentage differences (%)
Manual delineation	3.8100 ± 0.0388	3.8009 ± 0.0059	0.557	0.600	3.8054 ± 0.0269	0.71
Mimics segmentation	3.7602 ± 0.1117	3.7689 ± 0.0646	0.166	0.871	3.7645 ± 0.0871	2.31
Graph cut method	3.7790 ± 0.0445	3.7766 ± 0.0443	0.134	0.895	3.7778 ± 0.0434	1.45

* n = 6 (n = 12 in total MV) for manual delineation and Mimics segmentation, *n* = 12 (*n* = 24 in total MV) for graph cut method, because every observer calculated MV once for 6 test data by manual and Mimics method, but two times by graph cut method

#### Fat volume and variability calculation

The intraobserver and interobserver variability of segmentation methods were shown in Table 2. ICC of graph cut method were ranged from 0.996–0.999, indicating highly good inter- and intra-observer agreements. The fat volume variation of six phantoms calculated by graph cut method showed a conformable increasing consistent with the previously measured volume of butter. The absolute value differences between the test groups variation and ground truth of right orbit fat were calculated as 0.0482 ± 0.0301 cm^3^ in manual delineation, 0.3861 ± 0.2320 cm^3^ in Mimics segmentation and 0.0493 ± 0.0350 cm^3^ in graph cut segmentation. There was no significant difference in the comparison of manual delineation and graph cut (*p* = 0.960, paired t test) but significantly different between Mimics and graph cut (*p* = 0.031, paired t-test). The minimum volume variation in ground truth was 0.1cm^3^, and the corresponding graph cut measured value was 0.1101 ± 0.0158 cm^3^. The result showed the graph cut method could examine small volume changes minimum to 0.1cm^3^.

**Table 2:** The intraobserver and interobserver variability of segmentation methods (Intraclass Correlation Coefficient, ICC) and comparison of three different segmentation methods for FV calculation of orbital phantom data

Sector	Right Orbit		Left Orbit	
	ICC	*P* Value	ICC	*P* Value
Intraobserver				
OA Graph cut	0.997	< 0.01	0.999	< 0.01
OB Graph cut	0.999	< 0.01	0.998	< 0.01
Interobserver				
Graph cut (OA1-OB1)	0.996	< 0.01	0.998	< 0.01
Graph cut (OA1-OB2)	0.999	< 0.01	0.999	< 0.01
Manual	0.999	< 0.01	0.999	< 0.01
Mimics	0.879	< 0.05	0.993	< 0.01
3 Methods				
Manual - Graph cut	0.995	< 0.01	0.999	< 0.01
Mimics - Graph cut	0.876	< 0.05	0.988	< 0.01
Manual - Mimics	0.828	< 0.05	0.989	< 0.01

ICC outcome values: 0, no agreement; 1, complete agreement

#### Efficiency of the segmentation methods

**Fig. 3:**
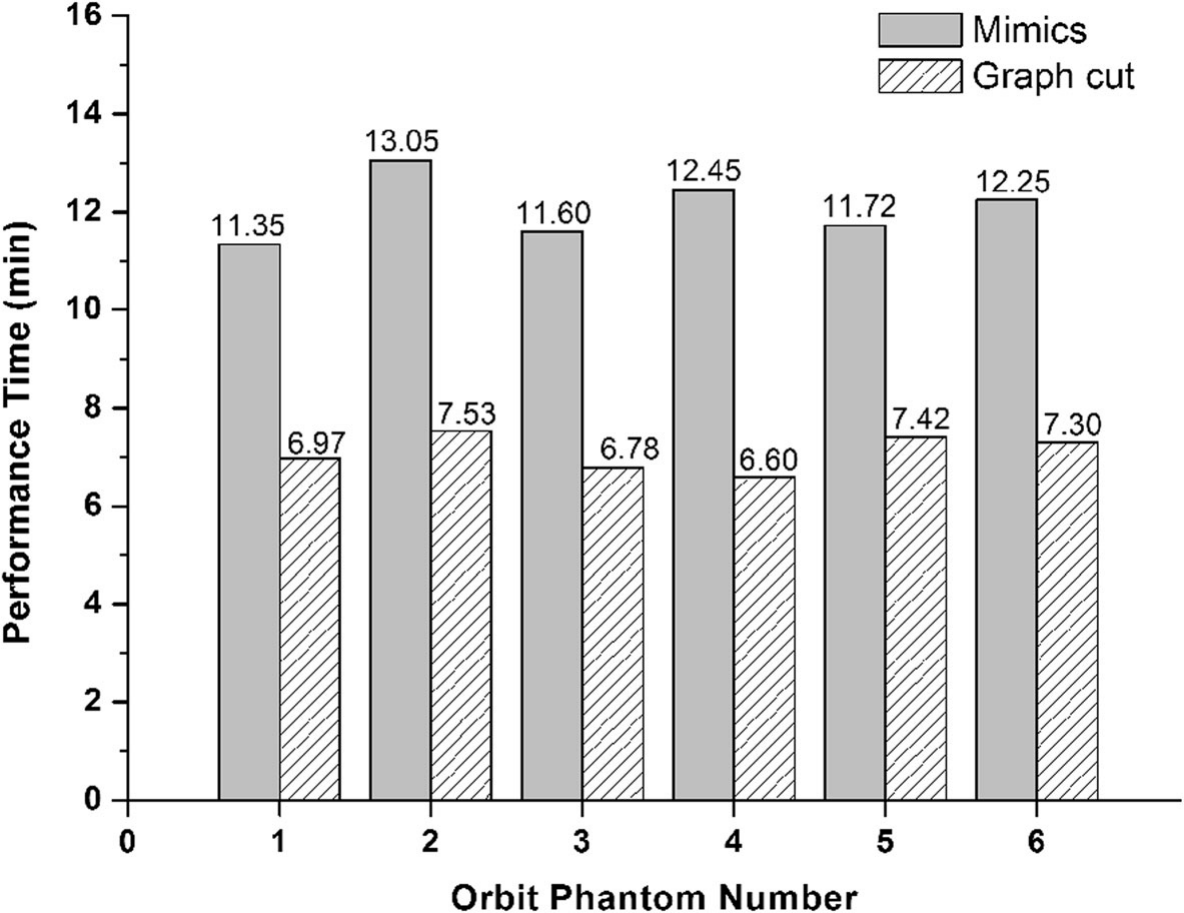
The performance time of different segmentation methods for orbital phantoms. The performance time was measured from the beginning of users defining the target tissue to the end of final result output, including both program automatic segmentation and slight manual modification

The performance time required for manual delineation of the six phantom data ranged from 3.38 to 4.80 h, with a mean time of 4.07 ± 0.52 h. The mean performance time for Mimics segmentation and graph cut segmentation were 12.07 ± 0.63 min and 7.10 ± 0.37 min respectively. The graph cut method took significantly less time than the Mimics segmentation needed to perform the same tasks (Fig. 3, *p* = 0.000, independent t test). As the performance time of manual delineation was enormously longer than image processing methods, the data were not shown in Fig. 3.

### Volume calculation and 3D reconstruction of orbital soft tissue after enucleation surgery

#### Normal orbit segmenting and calculating evaluation

**Fig. 4:**
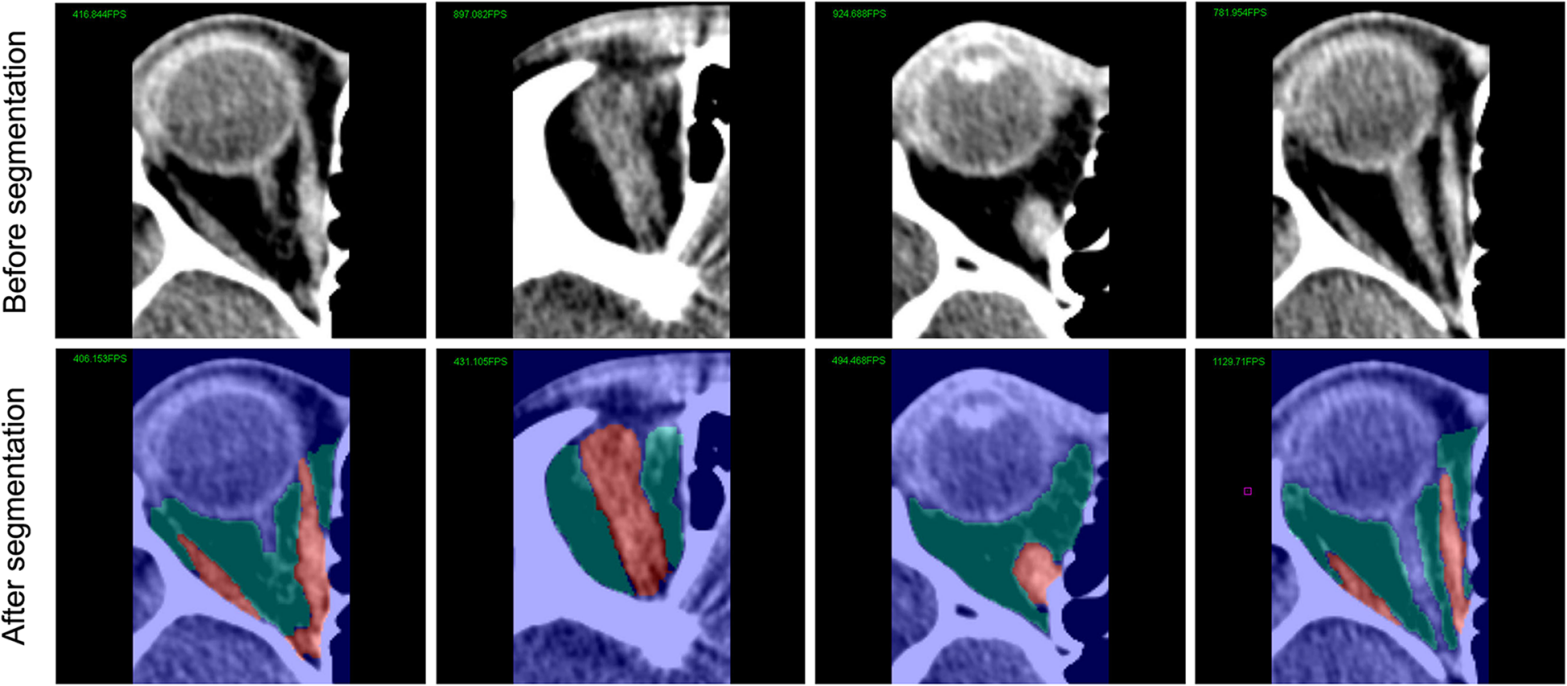
Segmentation results of graph cut method in real human normal orbital CT images. The top four graphs show different slices of normal orbital CT image of same patient. The bottom four graphs show segmentation results of graph cut method in these slices. Blue region represents intraorbital fat and red region represents extraocular muscle. The boundary of muscle and fat tissue is clear without obvious error or tissue omission

The graph cut method produced a receivable result that could perform segmentation in all the 3D slices and primarily identify muscle and fat tissue in the normal human orbit (Fig. 4). When performing muscle segmentation, there were many similar gray value pixels that corresponded to the brain, skin and other tissue outside the orbit that should not be included. It was difficult for Mimics thresholding segmentation to separate extraocular muscle specifically, and this approach might therefore need lots of manual assistance. For this reason, we did not use Mimics software to segment extraocular muscle tissue in this study.

**Fig. 5:**
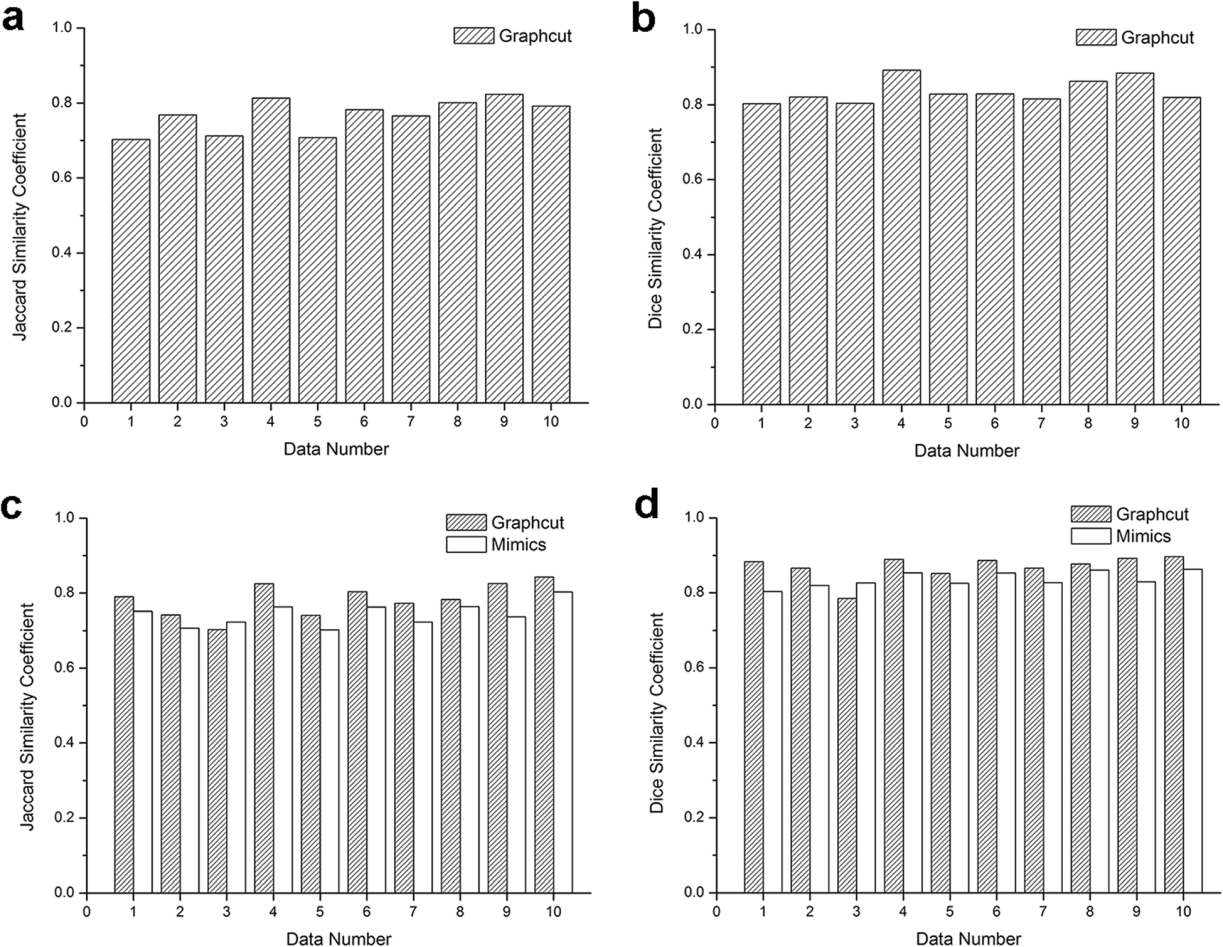
Jaccard similarity (JS) and Dice coefficient (DC) value of graph cut method and Mimics software for ten patients’ normal extraocular muscle and orbital fat segmentation. **a** and **b** are the JS and DC result of graph cut method for the extraocular muscle segmentation respectively. **c** and **d** respectively show the JS and DC comparison of graph cut method and Mimics software for intraorbital fat tissue segmentation. JS and DC value 0 means the measurements are completely dissimilar with ground truth and that 1 means the measurements and ground truth are identical

The extraocular muscle volume was 3.3959 ± 0.3056 cm^3^. Both the intraobserver and interobserver results presented good agreements. The correlation coefficients ranged from 0.807–0.968 (Table 3). The average JS and DC were 0.767 ± 0.045 and 0.836 ± 0.032 respectively (Fig. 5a, b).

**Table 3:** The intraobserver and interobserver intraclass correlation coefficient of graph cut segmentation for real human normal orbital MV and FV by the two observers

	OA1-OA2	OA1-OB	OA2-OB
MV	0.955^*^	0.807^*^	0.929^*^
FV	0.968^**^	0.937^*^	0.973^**^

* *p* < 0.05, ** *p* < 0.01 (*p* < 0.05 is considered to be statistically significant)

In fat tissue segmentation, JS value was 0.783 ± 0.044 for the graph cut method and 0.741 ± 0.032 for the Mimics software, which was significantly smaller than graph cut (*p* = 0.024, t-test). DC value of graph cut method was 0.869 ± 0.003 and 0.836 ± 0.020 for Mimics software segmentation, which was also significantly smaller than graph cut (*p* = 0.007, t-test). The results were shown in Fig. 5c, d respectively. The intraorbital fat volume was 10.9942 ± 1.0432 cm^3^ calculated by graph cut method.

#### Orbital soft tissue segmentation and reconstruction after enucleation surgery

**Fig. 6:**
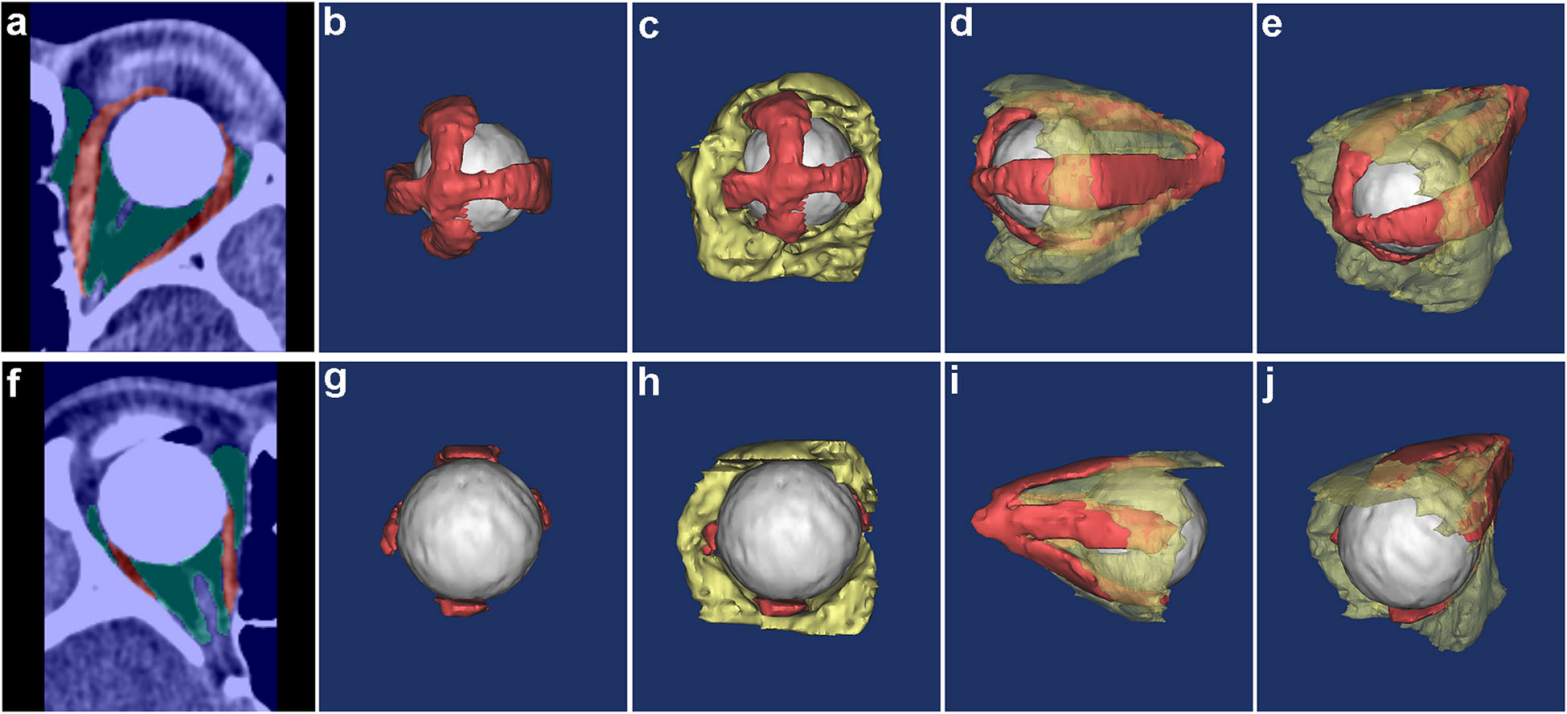
Graph cut segmentation result and 3D reconstruction of post-enucleation orbit following two different closure techniques. **a**-**e** are the segmentation and reconstruction results of one patient with his left eye enucleated and underwent rectus end-to-end suturing HA prosthesis implantation. **a** shows graph cut result on one slice of his orbital ROI CT images. **b**-**c** show the front view of reconstructed orbit with the intraorbital fat invisible or being displayed respectively. **d**-**e** show the lateral and top-oblique view of the structures. Fat tissue is semi-transparent in these two graphs. **f**-**j** are results of another patient with his right eye enucleated and underwent rectus orthotopic suturing HA prosthesis implantation

**Fig. 7:**
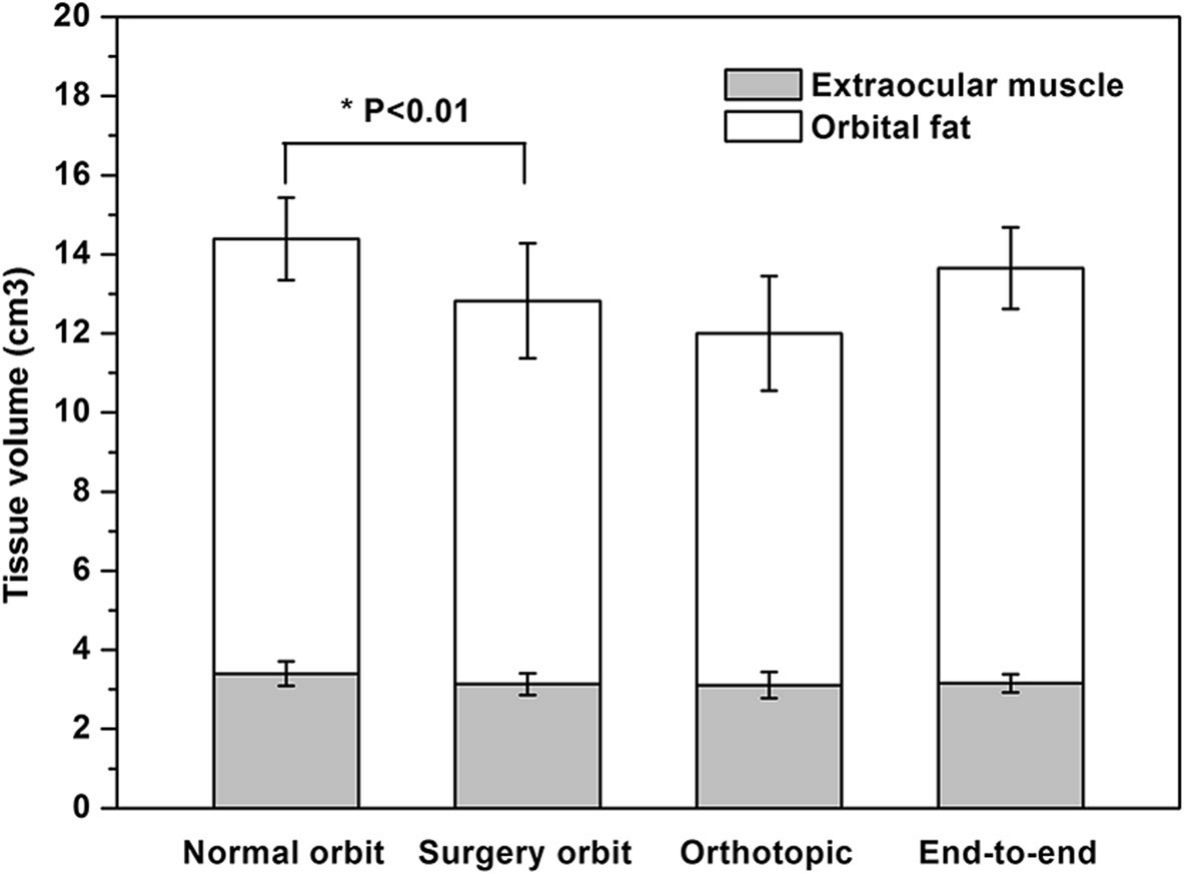
Extraocular muscle and intraorbital fat volume of patients’ normal and post-enucleation orbit

Interactive graph cut method could successfully segment and reconstruct the complex orbital soft tissue structures of the two different surgical methods. The visualization results were shown in Fig. 6. Muscle volume after enucleation was 3.1287 ± 0.2720 cm^3^, which was significantly smaller than normal side (*p* = 0.000, paired t test). Intraorbital fat volume was 9.6928 ± 1.4546 cm^3^ and significantly smaller than normal side as well (*p* = 0.001, paired t test). A comparison of the two closure procedures showed that the extraocular muscle volume was 3.1546 ± 0.2315 cm^3^ after rectus end-to-end suturing procedure and 3.10282 ± 0.3335 cm^3^ after rectus orthotopic suturing procedure. There was no significant difference between two procedures (*p* = 0.783, independent t test). Intraorbital fat volume was 8.8932 ± 1.4486 cm^3^ after rectus orthotopic suturing procedure, and this was smaller than the 9.6928 ± 1.4546 cm^3^ measured after rectus end-to-end suturing procedure. There was no significant difference between two procedures (*p* = 0.079, independent t test). The overall and different surgical procedures results were shown in Fig. 7.

## Discussion

In the present study, we proposed a novel graph cut algorism based method for soft tissue segmentation on CT image to assist the volume calculation and 3D reconstruction of intraorbital soft tissues. Our method was validated to have a good agreement with manual and commercial software measurement, and was more efficient than previous methods.

An accurate measurement and 3D reconstruction of intraorbital soft tissues is crucial to evaluation and treatment of orbital diseases. Traditionally, the regions of different soft tissues were marked by experienced clinicians on CT images and tissue volume was accordingly calculated. The measurements could be used as a reference standard to verify other methods [34]. However, the accuracy of this measurement is subject to different clinicians. With the development of medical image processing technique, some objective computer-assisted segmenting and calculating methods were used. Regensburg et al. calculated the volume of orbital soft tissue on CT image with the Mimics software, finding calculation differences of 0.7–2.2% in tissue volume and a < 5% difference between different experienced observers [2, 14]. Our study used the optimized graph cut segmentation method to identify extraocular muscles and intraorbital fat in CT image. The volume measurements were accurate compared to the known volume and manual measurements. Deference of MV volume was 0.58% compared with the known phantom volume, with the average graph cut calculated volume was 3.7778 ± 0.0434 cm3 and truth volume was 3.8 cm3, respectively. Moreover, the measurements obtained by two observers showed a high correlation (r = 0.996–0.999, *p* < 0.01), indicating a fair reliability and universality in different professional practiced doctors. In our method, users could obtain the calculation result through the automatic segmentation of computer program by simply labelling several sample lines in CT images. The performance greatly reduced the error caused by users’ subjective operation, and output an relatively objective result adjudged by computer algorithm based on unified segmentation criterion.

Thin slices and high spatial resolution of CT scan were benefit to perform more precise measurements and 3D reconstruction [35]. With the increasing data being processed, clinicians need a more efficient, fast-performing and accurate image processing method. Most of the reported methods for intraorbital soft tissue segmentation suffered with complex process and required much manual assistance. Many present medical image processing softwares identify various tissues due to the different tissue densities in medical image. However, tissue densities of orbital fat, extraocular muscle and other connective tissues were similar in CT scan, which increase the difficulty of automatic segmentation. Kim et al. measure the orbital soft tissues volume as evaluation index for Graves’ orbitopathy using Mimics software. During the target tissue selection, extraorbital fat needs to be manually erased by doctors because it cannot be automatically distinguished from the intraorbital fat by software [36]. In our method, the graph cut algorithm can take both CT value and tissue continuity into consideration. This enables the image processing program to automatically identify different tissues even with similar density during segmentation, thus reducing manual intervention and improve operational efficiency. The mean performance time of our graph cut method was nearly half of the commercial Mimics software, and merely 2.3% of manual segmentation as shown in Fig. 3. Moreover, the segmentation process was performed smoothly on a common configurated computer. This makes this approach easy for clinicians to use in daily clinical work without having to purchase special equipment.

Changes in the volume or distribution of intraorbital soft tissue can be used to assess disease progression or therapeutic effects [37–39]. Volumetry of extraocular muscles and orbital fat was an important assessing index of the severity and treatment efficiency in orbital inflammatory disease [36]. In assessment of orbital fracture, an increase of 1 ml in orbital volume would result in an average of 1 mm of enophthalmos [37, 40–42]. Thus in our study, we also evaluated the minimum volume change of soft tissue measured by our graph cut method. The minimum volume variation that could be detected was 0.1 cm3. This measurement accuracy can helpful to achieve an accurate clinical evaluation.

In our previous study, we investigated the implant size and exposure rate of 234 cases who went through two different surgical techniques for enucleation: the rectus end-to-end suturing procedure and the rectus orthotopic suturing procedure [28]. In this study, on the basis of having validated the accuracy of orbital soft tissue segmentation by graph cut method, we further applied it to the reconstruction and evaluation of clinical complex orbital conditions after ocular enucleation with the above procedures. The soft tissue volume was significantly lower than that of the normal side. This result might due to the surgical excision or atrophy of the tissue after operation. A larger soft tissue volume was detected in the patients underwent rectus end-to-end suturing procedure than those underwent the rectus orthotopic suturing procedure. Although the testing samples enrolled in this study were small, the graph cut method could be used to assess a large sample investigation of soft tissue changes in enucleation and other orbital disorders in future. In orbital segmentation of patients after enucleation surgery, the graph cut method presents good results in correctly identifying muscle and fat tissue by primary segmentation (Fig. 6), indicating a good potential of clinical application.

## Conclusion

In this study, an interactive graph cut method was developed to segment the orbital soft tissues in CT image. It is an accurate, reliable and efficient technique in segmentation, calculation and reconstruction of orbital soft tissues. This technique could be used to quantitatively assess the volume and three-dimensional relationships of multiple tissues under normal or pathological conditions, and it would therefore be useful in dynamic analyses and prediction of therapeutic effects and orbital reconstruction.

## Supplementary information

**Additional file 1.** Interactive graph cut segmentation for intraobital soft tissues. This file describes the graph cut and lazy snapping algorithm used in this study.

## Data Availability

The datasets and source code used and analyzed during the current study are available from the corresponding author on reasonable request.

## Abbreviations

3D: three dimensions
CT: computerized tomography
DC: Dice coefficient
FV: fat volume
HA: hydroxyapatite
ICC: intraclass correlation coefficients
JS: Jaccard similarity
MRI: magnetic resonance imaging
MV: muscle volume
ROI: region of interest
SD: standard deviation
TAO: hyroid-associated ophthalmopathy
