# Supplementary material for: An accurate interactive segmentation and volume calculation of orbital soft tissue for orbital reconstruction after enucleation
Source: BMC Ophthalmol. 2019 Dec 16;19:256. doi: 10.1186/s12886-019-1260-5 (PMC6916112; doi:10.1186/s12886-019-1260-5)
Supplement: Supplementary file 1 — Additional file 1. Interactive graph cut segmentation for intraobital soft tissues. This file describes the graph cut and lazy snapping algorithm used in this study. [file 12886_2019_1260_MOESM1_ESM.docx]

**Interactive graph cut segmentation for intraobital soft tissues**

In this study, the intraorbital tissue segmentation is automatically implemented by the computer following the principle of graph cut algorithm after defining the tissue samples by drawing marking lines (the operation method was described in the Methods section of the article).

Graph cut algorithm was first proposed by Boykov and Jolly in 2001. [20] It can utilize both regional and boundary information to achieve an optimized segmentation result. In this study, it is equivalent to finding the optimal solution of the following energy equation:

$$E\left( x \right)=\sum_{p\in P} E_{1}(x_{p})+\alpha\sum_{(p,q)\in E} E_{2}\left( x_{p}{, x}_{q} \right) (1)$$

where *E_1_* is a region term which means the cost of pixel *p* labelling as the target tissue or background, and related to the CT gray value of the pixel. *E_2_* is a boundary term which means the cost of the labels of neighboring pixels *p* and *q* as the same tissue or not, and related to both the position and gray value of the two pixels. Furthermore, based on the sample defined by lazy snapping, [30] *E_1_* and *E_2_* in equation (1) can be further expressed as follows:

$$E_{1}\left( x_{p} \right)=\left\{ \begin{matrix} \left( 1-x_{p} \right)\cdot K, if p\epsilon F \\ x_{p}\cdot K, if p\epsilon B \\ \frac{d_{p}^{F}+(1-x_{p})\cdot d_{p}^{B}}{d_{p}^{F}+d_{p}^{F}}, if p\epsilon U \end{matrix} \right. (2)$$

$$E_{2}\left( x_{p},x_{q} \right)=\frac{1}{\left\| V_{p}-V_{q} \right\|^{2}+1} (3)$$

where *F*, *B* and *U* are the foreground, background and unknown regions, respectively. Formula *d* means the minimum distance from pixel *p* to foreground/background clusters. And *V* indicates the CT gray value gradient between neighboring pixels *p* and *q*. Each pixel in the image can be classified into its belonging tissue region through the running of the algorithm, and ultimately make it possible to complete the segmentation of different tissues.
